# Phase Shift from a Coral to a Corallimorph-Dominated Reef Associated with a Shipwreck on Palmyra Atoll

**DOI:** 10.1371/journal.pone.0002989

**Published:** 2008-08-20

**Authors:** Thierry M. Work, Greta S. Aeby, James E. Maragos

**Affiliations:** 1 U. S. Geological Survey-National Wildlife Health Center, Honolulu Field Station, Honolulu, Hawaii, United States of America; 2 University of Hawaii, Hawaii Institute of Marine Biology, Kaneohe, Hawaii, United States of America; 3 U. S. Fish & Wildlife Service, Pacific Islands Refuges, Honolulu, Hawaii, United States of America; Monterey Bay Aquarium Research Institute, United States of America

## Abstract

Coral reefs can undergo relatively rapid changes in the dominant biota, a phenomenon referred to as phase shift. Various reasons have been proposed to explain this phenomenon including increased human disturbance, pollution, or changes in coral reef biota that serve a major ecological function such as depletion of grazers. However, pinpointing the actual factors potentially responsible can be problematic. Here we show a phase shift from coral to the corallimorpharian *Rhodactis howesii* associated with a long line vessel that wrecked in 1991 on an isolated atoll (Palmyra) in the central Pacific Ocean. We documented high densities of *R. howesii* near the ship that progressively decreased with distance from the ship whereas *R. howesii* were rare to absent in other parts of the atoll. We also confirmed high densities of *R. howesii* around several buoys recently installed on the atoll in 2001. This is the first time that a phase shift on a coral reef has been unambiguously associated with man-made structures. This association was made, in part, because of the remoteness of Palmyra and its recent history of minimal human habitation or impact. Phase shifts can have long-term negative ramification for coral reefs, and eradication of organisms responsible for phase shifts in marine ecosystems can be difficult, particularly if such organisms cover a large area. The extensive *R. howesii* invasion and subsequent loss of coral reef habitat at Palmyra also highlights the importance of rapid removal of shipwrecks on corals reefs to mitigate the potential of reef overgrowth by invasives.

## Introduction

The term “phase shift” as applied to coral reef ecosystems was first coined by Done [1] who used it to describe the change in reef biota from coral to macroalgae attributed to environmental degradation. Reasons cited for such phase shifts include overfishing, excessive nutrient input, predation by *Acanthaster plancii*, and depletion of major functional groups like fish and echinoid grazers [1]–[3]. Experimental manipulations have confirmed the important role that grazers play in keeping macroalgae from dominating reefs [4], and field observations of phase shifts from coral to algae subsequent to catastrophic disease events in echinoids confirm this [5].

Reports of overgrowth of degraded reefs by plants largely overshadow the less commonly cited phenomenon of overgrowth of reefs by other cnidaria such as anemones and corallimorphs [6]–[8]. Reasons for phase shifts from one type of cnidarian (coral) to another (anemones or corallimorphs) are speculative. Bleaching, damage by typhoon, overfishing, coastal development, and tourism were suspected as a cause of overgrowth of *Acropora* by the anemone *Condylactis* sp in Taiwan [6]. The corallimorpharian *Rhodactis rhodostoma* was most aggressively competitive in areas of Tanzanian reefs that had the highest levels of phosphate and man-made disturbance [7]. Restoring tropical reef ecosystems to a prior state subsequent to algal or cnidarian overgrowth can be a daunting task even when areas affected are small [9]. Therefore, understanding what drives such events may help prevention or aid mitigation.

Palmyra is a remote and comparatively pristine atoll located in the U.S. Line Islands in the central Pacific (05° N 162° W). Palmyra was extensively modified during WWII but abandoned shortly thereafter with sparse human influence since. In 2001, Palmyra became a National Wildlife Refuge. In 1991, a long line fishing vessel wrecked on the western shelf of the reef and remains grounded. The shipwreck was first examined by one of us (JEM) in 2004 where *R. howesii* were observed occurring at extremely low numbers. By 2005, *R. howesii* populations appeared to be expanding, and in 2006 permanent monitoring transects were established on the north and south sides of the wreck. Our objectives were to quantify the extent of the corallimorph invasion around the long line wreck and to document damage to corals at the gross and cellular levels.

## Materials and Methods

For towed diver surveys, a single boat operator (GSA) and diver on snorkel (TMW) made a straight course at constant speed from the ship toward the eight cardinal points of the compass (north, northwest, west, southwest, south, southeast, east, northeast). To ensure consistency, the same diver did all benthic surveys for corallimorphs. While transiting each course, a GPS reading was taken at 1-minute intervals and the diver visually estimated the average area of benthos covered by corallimorphs as high (>60% cover), medium (>30–60% cover), light (1–30% cover), or no impact (no corallimorphs visible). Each one minute interval covered ca. 60 linear meters. Linear transects surveyed extended 200 m to 1400 m from the ship. To ensure that the extent of impact was mapped as completely as possible, each course heading was continued until three consecutive 1-minute (60 m) observations revealed no corallimorphs or until it became too deep to see the benthos from the surface. Estimated depth of the areas surveyed ranged from 5 to 20 m, and the benthos was visible from the surface for all but the last point of two transects (south and southeast) where depth exceeded visibility (reef slope). We quantified corallimorphs within high-density areas approximately 5 m from the wreck by haphazardly placing 10 replicate 0.5 m^2^ quadrats on the bottom around the ship and counting all polyps within each quadrat. The ship was measured from bow to stern with a 100 m surveyor's tape.

To document the mechanism of injury inflicted by corallimorphs on scleractinian corals, corals were photographed, and samples of corals and corallimorphs in contact areas (five *Acropora pulchra* and five *Montipora capitata*) were collected and fixed in Z-fix. Corals were decalcified, tissues embedded in paraffin, sectioned at 5 um, and stained with hematoxylin and eosin for microscopic examination [10].

To determine population densities of corals at risk from invasion, injury, and death by corallimorphs, benthic surveys were conducted at the ends of the eight major survey courses (N, NW, NE, E, SE, S, SW) in areas bereft of corallimorphs. At each site, two consecutive 25 m lines separated by 5 m were deployed along the axis of the eight survey courses. At 50 cm intervals, the benthic substrate underlying the tape measure was classified as live coral (identified to genus), hard substrate, macroalgae, or crustose coralline algae. Data were tallied and percent coral cover calculated.

**Figure 1:**
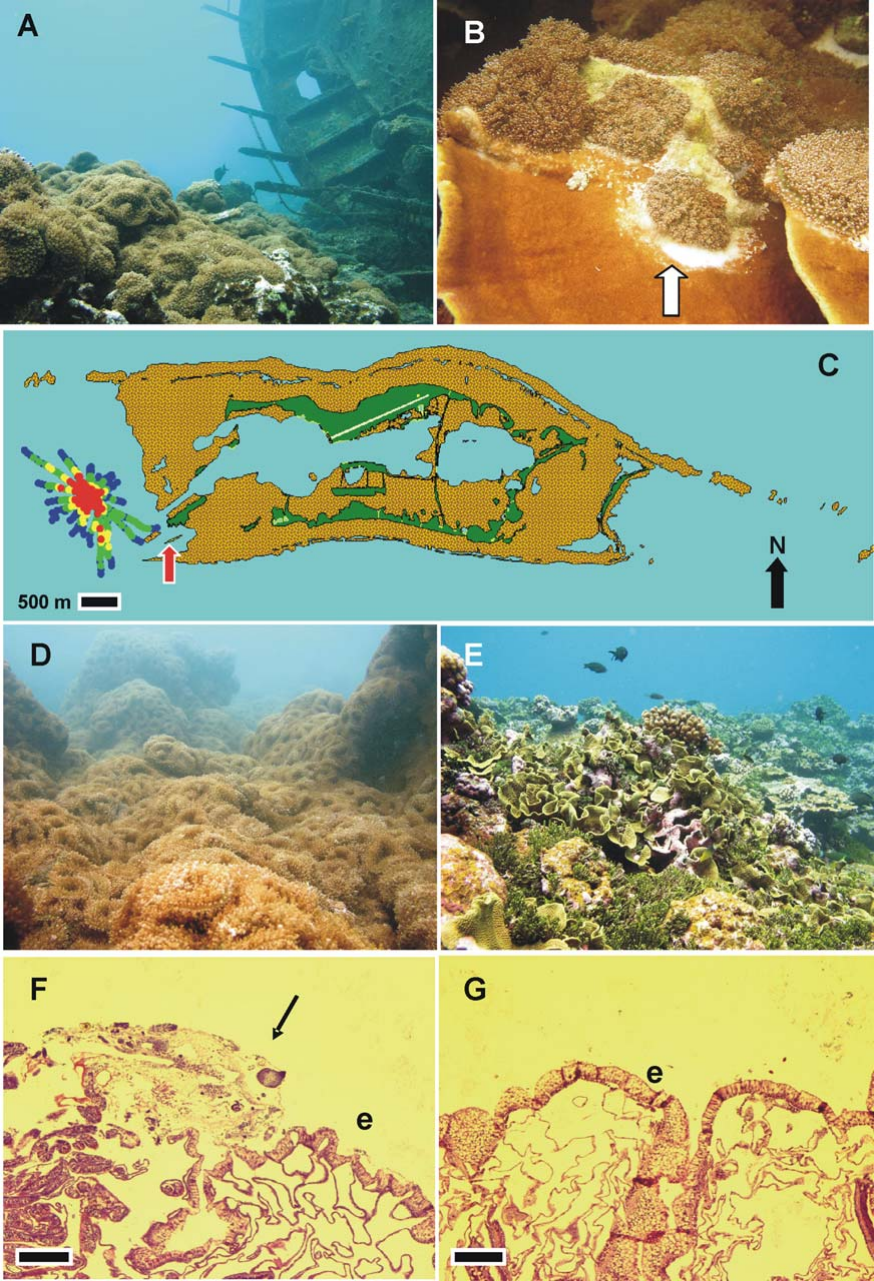
Invasion of the western shelf of Palmyra by corallimorphs from macro to micro scale. A) Shipwreck (right) and monoculture of *R. howesii* (left). B) Close up of *Montipora capitata* overgrown with *R. howesii*, note acute tissue loss where corallimorph has retracted (arrow). C) Extent of corallimorph infestation on western shelf of Palmyra Atoll NWR. Color codes dots correspond to estimated benthic cover of corallimorphs: red = high (>60%), yellow = medium (>30–60%), green = light (1–30%), blue = no visible corallimorphs. The ship is at the center of the red zone. Red arrow points to area where buoys were surveyed (Penguin Spit). The dark green area on the map represents emergent land with air strip (yellow line), and the brown area is submerged plateau surrounded by reef crest. D) Monoculture of *R. howseii* near ship. E) Unaffected reef. F) Photomicrograph of *Acropora* sp. with *R. howesii*-induced tissue loss manifesting tissue fragmentation and necrosis (arrow); e-epidermis, bar = 200 µm. G) Photomicrograph of normal *Acropora* sp.; e-epidermis, bar = 200 µm.

To confirm whether a numerical density gradient of corallimorphs may be associated with metal structures other than the ship, we quantified corallimorph abundance around three mooring buoys at Penguin spit, an area far removed from and not apparently impacted by the wreck (Fig. 1C). Individual organisms were counted within 30×1 m belt transects deployed along the axis of the 8 cardinal compass points (N, NW, NE, E, W, SE, SW, S) surrounding each buoy.

## Results

The long line vessel measures 37 m long and lies in about 8 m of water at an approximately 80 degree list on its port side with her stern pointing circa 30 degrees northeast. The hull appeared intact and no fuel was seen or smelled leaking from the ship, although a visible surface sheen of oil had been reported moving to the northwest of the wreck as recent as June 2005 by one of us (JEM). The immediate area around the vessel was littered with debris (batteries, metal poles, search light, pressured air cylinder, gloves, and other unidentified metal objects). The hull of the ship was bereft of organisms except algae and two colonies of *Pocillopora* sp. Around the ship, *R. howesii* covered the benthos overgrowing and directly competing with the few remaining corals (mainly *Montipora* sp., *Pocillopora* sp. and *Acropora* sp.) (Figs. 1A, B, D). On intact corals invaded by corallimorphs, diffuse areas of acute tissue loss were evident near enlarged marginal tentacles (Figure 1B). Mesenterial filaments were readily extended when corallimorphs were actively disturbed; however these structures were not normally seen during routine surveys. Histology of corallimorphs in contact with live corals revealed diffuse tissue loss and ablation of upper body wall of corals with necrosis of tissues (Fig. 1F, G).

**Figure 2:**
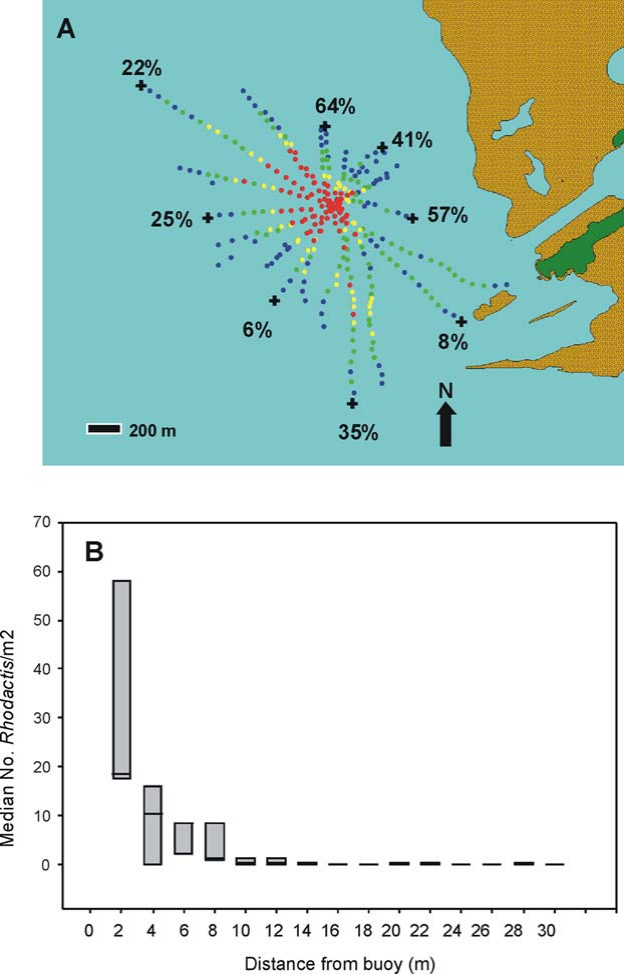
Details of corallimorph invasion near man-made objects. A) Extent of corallimorph infestation on western shelf of Palmyra Atoll NWR (close-up of Fig. 1C). Color codes correspond to estimated benthic cover of corallimorphs: red = high (>60%), yellow = medium (>30–60%), green = light (1–30%), blue = no visible corallimorphs. The ship is at the center of the red zone. Estimated percentage of coral cover at limits of corallimorph infestation. B) Box plot (5 and 95 percentile, median) of numbers of *Rhodactis*/m^2^ at 2 m intervals from 3 mooring buoys.

By the September 2007 survey, the invasion of the corallimorphs extended 2100 m at its widest point (Fig. 1C). Corallimorphs were present at distances from the ship ranging from 200 m to 1100 m (mean-590±300 m). The estimated coverage of the entire corallimorph-infested region was 1.0×10^6^ m^2^ with the high, medium, and low density regions comprising 15% (0.14×10^6^ m^2^), 26% (0.26×10^6^ m^2^) and 59% (0.60×10^6^ m^2^), respectively. Average density of corallimorph polyps within the high density area surrounding the wreck was 288 (SD±47) individuals per m^2^. Based on estimated area coverage of the infestation, estimated numbers of individual polyps surrounding the ship ranged from 52×10^6^ to 141×10^6^. Coral cover in areas at the periphery of the affected zone (populations of corals at risk of overgrowth) ranged from 6 to 64% with the highest (> = 25%) found in all but the southwest, northwest, and southeast sectors (Fig. 2A). Physical characteristics of the populations at risk ranged from mainly coral rubble to a diverse assemblage of stony coral species dominated by the genera *Montipora, Pavona,* and *Pocillopora* (Fig. 1E) Corallimorphs were also found surrounding three metal mooring buoys situated away from the impact of the wreck, and there was again a decrease in numbers of corallimorphs with increasing distance from buoys (Fig. 2B).

## Discussion

We report for the first time the potential of enhanced corallimorph spread associated with a shipwreck and the subsequent damage to coral reefs from corallimorph invasion. The high densities of *R. howesii* found radiating from the shipwreck were anomalous as prior surveys of reefs around Palmyra revealed this organism to be generally rare to absent except at the immediate vicinity of the wreck site [11]. Qualitative and quantitative surveys around the ship in 2005–2006 by one of us (JEM) found the corallimorph invasion to extend only approximately 50–100 m from the ship and coral cover to be 30% at the permanent site north of the shipwreck located at the outer edge of the corallimorph invasion. In contrast, we found corallimorphs extending up to 1100 m away from the ship with less than 1% coral cover near the shipwreck. This suggests a rapid population explosion of *R. howesii* culminating in the overgrowth of over 1×10^6^ m^2^ of reef by mid 2007. This area (ca. 2100×700 m) is the largest extent of corallimorph invasion of a reef reported to date.

Corallimorpharians have several life history traits which allow them to rapidly monopolize patches of shallow substrate in tropical habitats. They are competitively superior to some coral species and possess anatomic structures such as elongated marginal tentacles that allow them to kill competing scleractinian corals [12], [13]. When the corallimorpharian, *Corynactis californica*, was placed in contact with the reef coral *Astrangia lajollaensis*, 78 out of 80 interactions resulted in coral death [14], [15]. Additionally, corallimorpharians have three different modes of clonal replication (fission, pedal laceration and budding) that allow comparatively rapid monopolization of space on the reef [16]. In the Red Sea, *Rhodactis rhodosoma* was found to triple the occupied surface of the benthos each year allowing them to dominate space on a reef flat following a catastrophic low-tide disturbance [13].

Although the Palmyra shipwreck and associated oil spill may have opened up space on the reef for initial recruitment of *R. howesii*, corals in the immediate vicinity of the ship did not show microscopic morphology indicative of oil spill toxicity [17], and we saw no evidence of oil near the ship during our investigations. Rather, lesions were characterized by tissue necrosis associated with contact of marginal tentacles from *R. howesii* and attendant invasion by fungi and algae. In sum, *R. howesii* has both anatomic and life history traits that allow it to aggressively compete successfully for the benthos.

The subsequent extent of the area dominated by corallimorphs in this study was much greater than other studies where corallimorphs rapidly invaded damaged areas but to a much more localized extent [14], [15]. This may be due to some substance leaching from the ship such as dissolved iron [18]. Iron makes up the major component of steel and other ferrous metals in mooring buoys and ships, and is known to be a limiting resource for many marine organisms [18], [19]. Iron is an essential trace element for algal growth and nitrogen fixation [20], and equatorial and south Pacific oceanic waters are extremely low in available iron [21]. Phytoplankton productivity in oceanic waters is also limited by iron availability [22]–[24], and iron can be a limiting nutrient for primary producers on coral reefs including some algae (zooxanthellae) in symbiotic associations with soft corals and clams [25]. *Rhodactis howesii* also contain symbiotic zooxanthellae [26], accordingly, metal objects may also stimulate its growth.

Supporting this hypothesis is our finding of a numerical gradient of corallimorphs surrounding not only the ship but also metal buoys (structures bereft of fuel oil) suggesting that iron or some other component of the metal objects could be enhancing corallimorph growth perhaps through enrichment of their symbiotic algae (zooxanthellae) [26]. Patches of *R. howesii* have also been found at sites containing metal ship debris including historical ship landings at Baker and Howland Islands [27], and *R. howesii* was recently found appearing in patches at Kingman Reef in the vicinity of a fishing vessel which washed on the reef in mid 2007 (JEM). Our data are only observational, but the iron enrichment hypothesis deserves further exploration. Other than direct mechanical damage, few reports exist on the secondary effects of shipwrecks on reefs. One example is overgrowth of cyanobacteria on a reef at Rose Atoll (American Samoa) secondary to a ship grounding [28].

The extensive *R. howesii* invasion and subsequent loss of coral reef habitat at Palmyra Atoll and the potential association of corallimorph invasions with metal objects should serve as a clarion call to managers dealing with large metal objects such as wrecks on reefs. This will be especially relevant at remote low reef islands and atolls such as Palmyra where dissolved iron concentrations may be extremely limiting. The major sources of iron for the oceans are from wind-blown terrestrially derived dust [29] or in coastal regions, from rivers and terrestrial run-off [30]. Islands closer to continental landmasses or reefs at high islands where iron may be available from chemical erosion of volcanic soils may be less vulnerable to impacts of iron enrichment associated with metal objects. If removal of the shipwreck is not possible, regular systematic monitoring of the benthos for invasive organisms, including cnidarians, should be done to verify the rate of spread to support management actions such as removal of the shipwreck.

Although the generality of this phenomenon at other reefs awaits confirmation, in the case of Palmyra, the *R. howesii* infestation is beginning to reach catastrophic proportions. Given the ability of *Rhodactis* sp. to rapidly reproduce [14], [15], managers are now facing the possibility that even with removal of the ship sheer reproductive capacity of *R. howesii* action may continue to fuel its spread along the western reef shelf of Palmyra. Removal of the organism also promises to be a daunting task. Manual removal is labor intensive and is likely to spread reproductive fragments of the organism [14], [15]. Consideration should be given to chemically sterilizing the benthos, at least in densely affected areas, as was done to remove the invasive algae *Caulerpa* in California [9]. However, the efficacy of such interventions in a highly porous limestone substrate may be limited. Such dilemmas emphasize the critical importance of acting sooner than later to address shipwrecks on coral reefs.
